# Association between cardiovascular risk-factors and venous thromboembolism in a large longitudinal study of French women

**DOI:** 10.1186/s12959-021-00310-w

**Published:** 2021-08-21

**Authors:** C. J. MacDonald, A. L. Madika, M. Lajous, M. Canonico, A. Fournier, M. C. Boutron-Ruault

**Affiliations:** 1 INSERM (Institut National de la Santé et de la Recherche Médicale) U1018, Center for Research in Epidemiology and Population Health (CESP), Institut Gustave Roussy, Villejuif, France; 2 Université Paris-Saclay, Université Paris-Sud, Villejuif, France; 3 Université de Lille, CHU Lille, EA 2694 - Santé publique : épidémiologie et qualité des soins, F-59000 Lille, France; 4 Center for Research on Population Health, INSP (Instituto Nacional de Salud Pública), Cuernavaca, Mexico; 5 Department of Global Health and Population, Harvard T.H. Chan School of Public Health, Boston, MA USA

**Keywords:** Venous thromboembolism, Pulmonary embolism, Epidemiology, Prospective study

## Abstract

**Background:**

Previous studies have shown conflicting results regarding the influence of cardiovascular risk-factors on venous thromboembolism. This study aimed to determine if these risk-factors, i.e. physical activity, smoking, hypertension, dyslipidaemia, and diabetes, were associated with the risk of venous thromboembolism, and to determine if these associations were confounded by BMI.

**Methods:**

We used data from the E3N cohort study, a French prospective population-based study initiated in 1990, consisting of 98,995 women born between 1925 and 1950. From the women in the study we included those who did not have prevalent arterial disease or venous thromboembolism at baseline; thus 91,707 women were included in the study. Venous thromboembolism cases were self-reported during follow-up, and verified via specific mailings to medical practitioners or via drug reimbursements for anti-thrombotic medications. Hypertension, diabetes and dyslipidaemia were self-reported validated against drug reimbursements or specific questionnaires. Physical activity, and smoking were based on self-reports. Cox-models, adjusted for BMI and other potential risk-factors were used to determine hazard ratios for incident venous thromboembolism.

**Results:**

During 1,897,960 person-years (PY), 1, 649 first incident episodes of thrombosis were identified at an incidence rate of 0.9 per 1000 PY. This included 505 cases of pulmonary embolism and 1144 cases of deep vein thrombosis with no evidence of pulmonary embolism. Hypertension, dyslipidaemia, diabetes, smoking and physical activity were not associated with the overall risk of thrombosis after adjustment for BMI.

**Conclusions:**

Traditional cardiovascular risk factors were not associated with the risk of venous thromboembolism after adjustment for BMI. Hypertension, dyslipidaemia and diabetes may not be risk-factors for venous thromboembolism.

**Supplementary Information:**

The online version contains supplementary material available at 10.1186/s12959-021-00310-w.

## Introduction

Venous thromboembolism (VTE) is a multifactorial condition resulting from the formation of blood clots in the deep veins (deep vein thrombosis, DVT), or their migration to the lungs (pulmonary embolism, PE). Mortality rate estimates for the European Union give 500,000 VTE-related deaths per year [1]. VTE is considered a major global health burden, and a leading cause of lost disability-adjusted life years [2]. Established risk factors for VTE include long hospitalisations, surgery [3], and cancers [4]. Family history of VTE, non-O blood groups, and treatments such as menopausal hormone therapy (MHT) also increase the risk of VTE [5].

It is currently poorly understood how cardiovascular risk-factors contribute to VTE risk. Many common cardiovascular risk factors have been associated with VTE in meta-analyses including obesity, type 2 diabetes, smoking, non-O blood group, and high triglyceride concentrations [6, 7]. However, these associations are heterogeneous between studies [8, 9]. For example a recent cross-sectional study observed a positive association between hypertension and VTE [10]. However, a meta-analysis found that blood pressure was inversely associated with the risk of VTE [11], and a prospective study utilising time-varying covariates found no association [12]. Previous studies have failed to account for female specific risk-factors for VTE, such as parity, and the use of MHT [9]. As BMI is a major risk-factor for many of these conditions, it is possible that previous estimates not accounting for BMI are biased, and that common CVD risk-factors are not directly associated with the risk of VTE.

In this work, we aimed to explore the relationship between cardiovascular risk factors and VTE, in a large prospective study of middle-age, and older women controlling for specific female risk-factors for VTE. The hypothesis was that any associations between arterial risk-factors and VTE were confounded by BMI.

## Methods

### Study cohort

The E3N (Etude Epidémiologique auprès de femmes de la Mutuelle Générale de l’Education Nationale (MGEN)) cohort study was set up in 1990, and included 98,995 women born 1925–1950 and affiliated to the MGEN, a health insurance plan for workers in the education system and their spouses. The objective was to identify risk factors for cancer and chronic conditions in women. In 1990, participants signed an informed consent form in accordance to the French National Commission for Data Protection and Privacy. Part of this study is the French component of the European Prospective Investigation into Cancer (EPIC) [13].

Participants were asked every 2–3 years to complete self-administered questionnaires to provide and update medical events and lifestyle (1990, 1992, 1993, 1995, 1997, 2000, 2002, 2005, 2008, and 2011). The baseline for the current study was the date the participants answered the 1992 questionnaire. Participants were excluded from this study if they had stroke, coronary heart disease, cancer, death (*n* = 6452), or VTE (*n* = 390) before 1992. We excluded 721 participants with no reported baseline body mass index (BMI). The final study population included 91,707 women.

### Assessment of venous thromboembolism cases

Non-fatal incident VTE cases were identified from self-reports in the follow-up questionnaires sent to the study participants after baseline. Before the 2008 questionnaire, participants were asked to report VTE events (without distinguishing between deep and superficial VTE) as yes/no, and the corresponding date, or pulmonary embolism (PE) and the corresponding date. From 2008 on, questionnaires were specific for DVT or PE, asking participants not to report superficial VTE. A flow-chart of case identification is presented in the supplementary material (sup. Fig. 1). All cases were considered validated, either by imaging procedures, or by reimbursement for anti-thrombotic medications following the event.

When participants reported VTE or PE events in the 2005 or preceding questionnaires, they were contacted via another letter, whereby they were asked to provide medical documents relating to the event. In addition, a questionnaire including information on potential predisposing factors (including surgeries, immobilisation over 8 days, cancers, long voyages on airplanes, or other factors) for thrombosis and characteristics of the event was sent to the medical doctors who followed these participants, permitting classification of the events as primary or secondary. To be validated, clinical events had to be diagnosed using an imaging procedure. PE was defined as the presence of either a positive pulmonary angiography or a positive helicoidal computed tomography or a high-probability ventilation/perfusion lung scan. DVT had to be diagnosed by use of compression ultrasonography or venography.

For DVT/PE identified from the 2008 and 2011 questionnaires, we confirmed that cases were associated with a relevant antithrombotic prescription (Heparin, Tinzaparin, Fragmin, Fraxiparin, Enoxaparin, Acénocoumarol, Fluindione, Warfarin, Dabigatran, Rivaroxaban, or Apixaban) at least twice in the year following the VTE using the MGEN database. Self-reported cases that were not validated with anti-thrombotic medications were not considered as valid cases. We cross-referenced self-reported VTE with self-reported hospital admissions, to determine if there was any associated cancer, immobilisation, hospitalisation, surgery (e.g. hip and knee surgeries), or trauma, which would indicate a secondary VTE. All other cases were considered a primary event with no obvious cause.

Fatal cases were identified from national death registers using International Classification of Diseases (ICD) -9 (codes 4151 and 4539) and ICD-10 (codes I26.0 and I26.9).

Amongst the 91,711 participants in the study, 1443 incident first cases of VTE were identified during follow-up (1992–2005) as previously described [14], and a further 1277 identified from in 2008 and 2011. Cases of superficial and upper extremity VT (*n* = 848) and cases for which the type could not be determined (*n* = 223) were not considered. As a result, 1649 incident cases of VTE consisting of 505 cases of PE and 1144 cases of DVT with no evidence of PE were considered.

### Assessment of cardiovascular risk factors

All cardiovascular risk factors considered were updated at each questionnaire cycle for this study.

During the study period, women were asked if they had received a diagnosis of hypertension that required treatment in all questionnaires. In 2004, a drug reimbursement database became available for women in the study. Previously we have observed an 82% positive predictive value/agreement from self-reports when cross correlating with the drug reimbursement database for antihypertensive medications. (Anatomical Therapeutic Chemical Classification System codes C02, C03, C07, C08, and C09).

Type-2 diabetes cases were based on self-reports, which were then validated through a specific questionnaire mailed to women having reported type-2 diabetes, confirming either elevated glucose concentration at diagnosis (fasting ≥7.0 mmol/l or random glucose ≥11.1 mmol/l), treatment with diabetes drugs, and/or fasting glucose or HbA1c ≥ 7%,(53.0 mmol/mol), respectively [15]. Cases occurring after 2004 were identified using the MGEN reimbursement database. All women reimbursed for glucose lowering medications at least twice in a given year were classified as having type-2 diabetes.

Dyslipidaemia was self-reported at baseline, and at all follow up questionnaires. Participants in the study were asked if they had received a diagnosis of abnormal cholesterol from their doctor, or if they required treatment to control their cholesterol. Participants who did not provide information on dyslipidaemia were classified as unknown. The use of lipid-lowering medications was accounted for from 2004 onwards, using the MGEN database. Prior to 2004, statin use was considered unknown. As a secondary analysis, due to potential effects of statins on VTE risk, we considered the combination of self-reported dyslipidaemia, as well as statin reimbursement as ‘treated dyslipidaemia’. Separate classes were also created for ‘reported dyslipidaemia and no statin reimbursement’ and ‘no reported dyslipidaemia and statin reimbursement’.

We assessed usual physical activity from questionnaires that included questions on the time spent walking (to work, shopping, and leisure time), cycling (to work, shopping, and leisure time), housework, and sports activities. Metabolic equivalents (METs) per week were estimated by multiplying the hourly average METs for each item based on values from the Compendium of Physical Activities [16] by the reported activity duration. Physical activity was split into three tertiles depending on the population distribution. Blood ABO group and smoking were based on self-reports, and at each questionnaire participants were classified as smoker, ex-smoker, or never smoker.

### Assessment of adjustment variables

Self-reported height and weight were used to calculate body mass index (BMI), defined as weight (kg) divided by squared height (m^2^). In the cohort, self-reported anthropometry is considered reliable from a validation study [17].

Use of MHT was assessed using a booklet containing photos of all types of oestrogens and progestogens, as previously described [14]. Age at, and type of menopause was defined as either (in decreasing order of priority) age at last menstrual period, age at bilateral oophorectomy, self-reported age at menopause, age at start of MHT, or the age at the start of menopausal symptoms. If unavailable, the median age at menopause for the cohort (51 years for natural menopause, 47 years for artificial menopause) was imputed. Women were considered menopausal at baseline if any of these events occurred before the start of follow-up. Parity was based on self-reports. Family history of cardiovascular disease (stroke or coronary disease in either parent) was based on self-reports. Incident fractures, cancers, heart attacks, and strokes occurring during follow-up were based on cases validated by specific questionnaires to the women and their practitioners.

### Statistical analysis

As risk-factor status can change over time, we updated values in modelling using information from follow-up questionnaires, similar to the method used by Wattanakit *et at* [12], i.e. the dataset contained multiple rows for each participant. If a participant reported hypertension, diabetes, or dyslipidaemia in one questionnaire, they were considered to have this condition for the remainder of follow-up.

Outcomes considered were the first VTE, then the first PE, or DVT; and then models considered the status of the event, i.e. primary VTE, and secondary VTE. In order to account for competing events when considering the specific types of VTE, the other types of VTE were censored from the analysis at the time of the competing event.

Potential confounders were selected with the help of directed acyclic graphs (supplementary Fig. 2). Risk-factors were assessed one by one, (i.e. we did not mutually adjust for hypertension, diabetes and dyslipidaemia), using Cox proportional hazard models with age as the time-scale in order to account for the effect of age. Models were initially assessed with age as the time scale (model 1), then on statin use (yes/no), for education level (high-school/no high-school/university), parity (0, 1, > 1), menopausal status (yes/no), ever use of MHT (yes/no), type of menopause (natural/artificial) (model 2), and finally BMI (model 3). Models were not mutually adjusted for the considered risk factors, in order to reduce the likelihood of introducing collider bias. Time at entry was the age at the beginning of follow-up (i.e., the age when the participants answered the questionnaire sent out in 1992); exit time was the age when participants were diagnosed with VTE, died (dates of death were obtained from the participants’ medical insurance records), were lost to follow-up, or reached the end of the follow-up period (December 31, 2011), whichever occurred first.

As VTE can be provoked by bone fractures, other cardiovascular diseases, or cancer, we also considered a model controlled for incident fractures (time dependent, yes/no), cancers (time dependent, yes/no), and both heart attack and stroke (time dependent, yes/no), during follow-up as sensitivity analysis. The next sensitivity analysis excluded cases occurring after 2005 that were not part of the mailing conducted to validate the VTE cases. Another sensitivity analysis mutually adjusted for comorbid conditions at baseline, i.e. when considering hypertension, diabetes and dyslipidaemia were adjusted on at baseline. Finally, in the case of associations which were unexpected or suspected to be due to bias, we considered models using only baseline variables to determine if this was due to the inclusion of collider variables during follow-up.

When considering blood-groups 7834 participants were excluded from this analysis due to missing data on blood group. As a hypothesis generating exercise, we wished to determine if the associations between these risk factors were consistent over blood-groups, which are a major risk-factor for VTE. As blood-group is a determinant of blood lipid levels [18], hypertension [19], diabetes [20], and coagulation factors including von Willibrand factor [21], we considered effect modification was plausible regarding dyslipidaemia, hypertension and diabetes.

Missing values (occurring in less than 5% of data) were imputed as the mean for continuous variables, and the median for categorical variables. During follow-up, if a value was missing, the previous value was imputed.

All statistical analyses were performed using R and R studio, with the ‘Survival’ package. A Bonferroni corrected *p*-value for statistical significance was 0.01. The proportional hazards assumption was assessed using the cox.zph function in R. A p-value is generated for the Person product-moment correlation between the scaled Schoenfeld residual, and the time transformation for each variable [22].

## Results

During 1,897,960 person-years (PY) (mean follow-up of 20.7 years), 1649 first incident episodes of VTE were identified at an incidence rate of 0.9 per 1000 PY.

Table 1 displays data at baseline, and after 10 years of follow-up. The cardiovascular risk profile of the study participants worsened during follow-up, although total physical activity increased. BMI increased during follow-up from a mean of 22.9 kg / m^2^ to 23.8 kg / m^2^. Hypertension prevalence increased from 35.2 to 44.4%, diabetes from 1.0 to 2.2%, and self-reported dyslipidaemia from 7.8 to 25.4%. Smoking prevalence decreased during follow-up from 14.5 to 11.0%. At 2004, approximately 25% of study participants had been reimbursed for statins.

**Table 1:** Participant demographics at the beginning of followup, and mid-way through the study period

	Status at 1992 (*n* = 91,711)	Status at 2002 (*n* = 88,302)
Dynamic variables		
Age (years)	51.1 (6.6)	61.4 (6.6)
Declared hypertension (%)	35.2	44.4
Treated hypertension (%)*	Unknown	26.7
Diabetes (%)	1.0	2.2
Declared dyslipidaemia (%)	7.8	25.4
Statin user (%)*	Unknown	25.1
Treated dyslipidaemia (%)*	Unknown	12.9
BMI (kg / m^2^)	22.9 (3.3)	23.8 (3.8)
BMI < 18.5 (%)	4.0	3.9
18.5–24.9 (%)	75.8	68.7
25.0–30.0 (%)	16.4	21.6
> 30.0 (%)	3.8	5.8
Physical activity (METs)	51.4 (30.2)	60.5 (37.6)
Physical activity < 34.3 METs (%)	33.1	33.5
Physical activity 34.3–57.8 METs (%)	33.0	32.1
Physical activity > 57.8 METs (%)	33.9	34.9
Current smoker (%)	14.5	11.8
Ex-smoker (%)	31.4	34.9
Never smoker (%)	54.1	53.3
Ever MHT use (%)	43.2	55.5
Static variables		
Education level (High-school / University (> = BAC + 4))	65.1 / 34.9	
Parity (number of pregnancies)	2.1	
Natural menopause (%)	88.0	
Family history CVD (%)	30.6	
Non-O blood group (%)	56.6	

*assessed in 2004

2488 participants died between 1992 and 2002

In unadjusted models, hypertension was associated with an increased risk of VTE (HR _HTA_ = 1.21 (1.10: 1.34)), but this was not consistent after adjustment for BMI (HR _HTA_ = 1.05 (0.95: 1.16)). Current smokers were associated with a lower risk of VTE in unadjusted models, but this was not observed after adjustment for BMI (HR _smokers_ = 0.87 (0.73: 1.03)). Mean BMI among smokers at baseline was 22.5 (3.3) kg / m^2^ compared to 22.9 (3.3) kg / m^2^ among never smokers. Diabetes, dyslipidaemia, and physical activity were not associated with the risk of VTE in unadjusted or adjusted models (Table 2). Neither treated hypertension, nor treated dyslipidaemia were associated with the risk of VTE (not tabulated).

**Table 2:** Hazard ratio and 95 % confidence intervals for cardiovascular risk-factors and the risk of VTE, and VTE subtypes

	All VTE				Pulmonary embolism				Deep vein thrombosis				Primary VTE				Secondary VTE			
	Cases	unadjusted	adjusted*	adjusted + BMI	Cases	unadjusted	adjusted*	adjusted + BMI	Cases	unadjusted	adjusted*	adjusted + BMI	Cases	unadjusted	adjusted*	adjusted + BMI	Cases	unadjusted	adjusted*	adjusted + BMI
physical activity T1	545	ref	ref	ref	166	ref	ref	ref	376	ref	ref	ref	163	ref	ref	ref	382	ref	ref	ref
physical activity T2	545	1.03 (0.91: 1.16)	1.00 (0.89: 1.12)	1.03 (0.92: 1.16)	160	1.02 (0.82: 1.26)	0.99 (0.80: 1.23)	1.04 (0.84: 1.29)	387	1.04 (0.90: 1.20)	1.01 (0.88: 1.16)	1.04 (0.90: 1.20)	156	0.97 (0.78: 1.20)	0.95 (0.76: 1.18)	0.97 (0.78: 1.21)	389	1.06 (0.72: 1.21)	1.02 (0.89: 1.18)	1.06 (0.92: 1.22)
physical activity T3	559	1.02 (0.90: 1.14)	0.98 (0.87: 1.10)	1.02 (0.90: 1.15)	179	0.98 (0.80: 1.21)	0.95 (0.77: 1.18)	1.01 (0.82: 1.25)	381	1.03 (0.90: 1.20)	0.99 (0.86: 1.14)	1.02 '0.89: 1.18)	113	0.70 (0.55: 0.89)	0.68 (0.54: 0.87)	0.71 (0.56: 0.90)	446	1.15 (1.00: 1.32)	1.10 (0.95: 1.26)	1.15 (0.99: 1.32)
Never smoker	946	ref	ref	ref	295	ref	ref	ref	652	ref	ref	ref	253	ref	ref	ref	693	ref	ref	ref
X smoker	557	0.97 (0.87: 1.08)	0.97 (0.87: 1.07)	0.94 (0.85: 1.05)	166	0.99 (0.82: 1.19)	0.98 (0.81: 1.18)	0.96 (0.70: 1.33)	392	0.96 (0.85: 1.09)	0.97 (0.85: 1.09)	0.95 (0.84: 1.07)	131	0.85 (0.69: 1.04)	0.86 (0.70: 1.06)	0.84 (0.68: 1.04)	426	1.02 (0.90: 1.14)	1.01 (0.89: 1.13)	0.98 (0.87: 1.11)
Smoker	146	0.80 (0.67: 0.95)	0.85 (0.71: 1.01)	0.87 (0.73: 1.03)	44	0.90 (0.66: 1.24)	0.93 (0.68: 1.28)	0.94 (0.78: 1.14)	100	0.76 (0.62: 0.94)	0.82 (0.67: 1.01)	0.94 (0.68: 1.03)	48	0.91 (0.66: 1.24)	0.97 (0.71: 1.33)	0.99 (0.73: 1.36)	98	0.75 (0.61: 0.93)	0.79 (0.64: 0.98)	0.82 (0.66: 1.01)
hypertension	827	1.21 (1.10: 1.34)	1.18 (1.07: 1.30)	1.05 (0.95: 1.16)	296	1.42 (1.18: 1.69)	1.41 (1.18: 1.69)	1.20 (0.99: 1.45)	532	1.13 (1.01: 1.27)	1.10 (0.98: 1.24)	0.99 (0.88: 1.12)	204	1.17 (0.97: 1.42)	1.13 (0.93: 1.38)	1.02 (0.84: 1.25)	623	1.23 (1.10: 1.38)	1.21 (1.08: 1.35)	1.06 (0.94: 1.20)
dyslipidaemia	354	0.92 (0.82: 1.03)	0.95 (0.84: 1.06)	0.90 (0.79: 1.01)	142	1.02 (0.83: 1.25)	1.05 (0.86: 1.29)	1.03 (0.84: 1.26)	212	0.84 (0.72: 0.98)	0.85 (0.73: 1.00)	0.83 (0.71: 0.97)	69	0.67 (0.51: 0.88)	0.69 (0.53: 0.90)	0.68 (0.52: 0.89)	285	0.99 (0.86: 1.13)	1.00 (0.87: 1.15)	0.98 (0.85: 1.12)
diabetes	36	0.98 (0.71: 1.37)	0.98 (0.71: 1.37)	0.73 (0.52: 1.02)	17	1.24 (0.76: 2.01)	1.26 (0.77: 2.04)	0.87 (0.53: 1.43)	19	0.83 (0.53: 1.31)	0.83 (0.52: 1.30)	0.63 (0.40: 1.00)	11	1.28 (0.70: 2.33)	1.24 (0.68: 2.27)	0.96 (0.52: 1.76)	25	0.89 (0.60: 1.33)	0.90 (0.61: 1.34)	0.66 (0.44: 0.98)

When considering VTE subtypes (Table 2), including PE, DVT, primary, and secondary VTE, hypertension showed a borderline positive association with PE (HR_hta_ = 1.20 (0.99: 1.45)) after adjustment for BMI, and was consistent when considering treated hypertension, but not untreated hypertension (HR_hta trt_ = 1.24 (0.99: 1.56), HR_hta untrt_ = 1.16 (0.93: 1.45)). An inverse association with the highest tertile of physical activity was observed for primary VTE (HR_T3_ = 0.71 (0.56: 0.90)) after adjustment for BMI. Dyslipidaemia was associated with a reduced risk of primary VTE (HR_dys_ = 0.68 (0.52: 0.89)), and DVT (HR_dys_ = 0.83 (0.71: 0.97)), after adjustment for BMI. When the dyslipidaemia and statin treatment variables were combined, ‘treated dyslipidaemia’ was not significantly associated with a reduced risk of primary VTE (HR _treated dys_ = 0.98 (0.84: 1.16), not tabulated), compared to participants reporting no dyslipidaemia or statin reimbursement. This was also observed for DVT (HR_treated dys_ = 0.85 (0.68: 1.05), not tabulated). Diabetes was associated with a reduced risk of secondary VTE (HR_diab_ = 0.66 (0.44: 0.98)), but only 25 cases occurred among participants with diabetes, and was not significant after Bonferroni correction.

Results were similar when controlling for incident coronary disease, stroke, and cancer (supplementary Table 1). Association between hypertension and PE were consistent, (HR _hta_ = 1.20 (1.00: 1.45)), and similarly for dyslipidaemia and DVT (HR _dys_ = 0.83 (0.71: 0.97)) and primary VTE (HR_dys_ = 0.68 (0.52: 0.89)). Similarly, results were comparable when considering only cases that were validated by imaging procedures (Table 3), although the effect estimate confidence intervals were wider due to the reduced number of cases. Association between hypertension and PE were borderline, but of the same magnitude (HR_hta_ = 1.22 (0.95: 1.56)), and similarly for dyslipidaemia and DVT (HR_dys_ = 0.86 (0.71: 1.04)). Associations between dyslipidaemia and primary VTE were consistent (HR_dys_ = 0.74 (0.56: 0.99)), as were associations between physical activity and primary VTE (HR_T3_ = 0.70 (0.54: 0.90)). When mutually adjusting for comorbid conditions at baseline, results were similar to those presented in the main analysis (data not tabulated).

**Table 3:** Sensitivity analysis including cases occurring until 2005 which were strictly validated via mailing

	All VTE		Pulmonary embolism		DVT		Primary		Secondary	
	Cases	adjusted HR*	Cases	adjusted HR*	Cases	adjusted HR*	Cases	adjusted HR*	Cases	adjusted HR*
physical act T1	413	ref	97	ref	316	ref	154	ref	259	ref
physical activity T2	422	1.04 (0.90: 1.19)	97	1.05 (0.79: 1.39)	325	1.03 (0.88: 1.21)	148	0.97 (0.77: 1.22)	274	1.08 (0.91: 1.28)
physical activity T3	394	0.98 (0.85: 1.12)	96	1.00 (0.75: 1.33)	298	0.97 (0.83: 1.14)	107	0.70 (0.54: 0.90)	287	1.14 (0.96: 1.34)
Never smoker	721	Ref	172	ref	549	ref	241	ref	480	ref
X smoker	383	0.92 (0.82: 1.05)	88	0.93 (0.72: 1.20)	295	0.92 (0.80; 1.07)	120	0.87 (0.71: 1.09)	263	0.95 (0.82: 1.10)
smoker	125	0.89 (0.74: 1.08)	30	0.99 (0.67: 1.47)	95	0.86 (0.69: 1.07)	48	1.05 (0.77: 1.44)	77	0.81 (0.64: 1.04)
hypertension	583	1.02 (0.91: 1.15)	162	1.22 (0.95: 1.56)	421	0.97 (0.84: 1.11)	191	0.96 (0.78: 1.18)	392	1.05 (0.91: 1.22)
dyslipidaemia	189	0.86 (0.73: 1.01)	52	0.87 (0.64: 1.19)	137	0.86 (0.71: 1.04)	58	0.74 (0.56: 0.99)	131	0.93 (0.76: 1.13)
diabetes	21	0.71 (0.46: 1.11)	6	0.68 (0.30: 1.54)	15	0.73 (0.44: 1.22)	10	1.01 (0.54: 1.92)	11	0.56 (0.31: 1.03)

*adjusted on education level, statin use, menopause, MHT use, parity, type of menopause, family history of CVD and BMI

Associations between hypertension, dyslipidaemia and VTE differed slightly depending on ABO blood group (Table 4). Hypertension was associated with a higher risk of primary VTE amongst O-group participants (HR_HTA_ = 1.56 (1.07: 2.27)), and a non-significant increased risk of PE (HR_HTA_ = 1.21 (0.88: 1.68)). Dyslipidaemia was associated with a reduced risk of VTE amongst O-group participants (HR_DYS_ = 0.78 (0.63: 0.98), Table 3), with similar associations for all types of VTE (Table 4), but these associations were not consistent when considering the ‘treated dyslipidaemia’ variable, and showed no significant deviation from HR = 1 (not tabulated).

**Table 4:** Hazard ratio and 95% confidence intervals for cardiovascular risk factors and specific VTE type in strata of blood ABO group for metabolic conditions

	p-interaction*	All VTE	Pulmonary Embolism	Deep vein thrombosis	Primary	Secondary
Group O						
hypertension	0.01	1.14 (0.95: 1.38)	1.21 (0.88: 1.68)	1.10 (0.88: 1.39)	1.56 (1.07: 2.27)	1.03 (0.83: 1.28)
dyslipidaemia	0.78	0.70 (0.58: 0.85)	1.10 (0.78: 1.53)	0.60 (0.44: 0.83)	0.67 (0.42: 1.09)	0.82 (0.64: 1.06)
diabetes	0.82	0.68 (0.36: 1.29)	0.55 (0.20: 1.50)	0.79 (0.35: 1.80)	1.31 (0.47: 3.66)	0.51 (0.23: 1.17)
Group non-O						
hypertension	–	0.97 (0.85: 1.11)	1.05 (0.82: 1.36)	0.95 (0.81: 1.10)	0.78 (0.60: 1.01)	1.05 (0.90: 1.23)
dyslipidaemia	–	0.78 (0.69: 0.88)	0.78 (0.63: 0.97)	0.78 (0.67: 0.90)	0.63 (0.48: 0.83)	0.83 (0.72: 0.95)
diabetes	–	0.78 (0.51: 1.18)	1.19 (0.66: 2.15)	0.56 (0.31: 1.01)	1.02 (0.47: 2.19)	0.71 (0.43: 1.16)

* interaction with all-VTE

* adjusted on education level, statin use, menopause, MHT use, parity, type of menopause, family history of CVD and BMI

## Discussion

In this large cohort of women, hypertension, dyslipidaemia, and diabetes were not associated with the risk of all-VTE after adjustment for BMI. We observed negative associations between physical activity and the risk of primary VTE, and potential negative associations between dyslipidaemia and primary VTE.

In this study, no evidence for associations between cardiovascular risk-factors and all-VTE was observed, or disappeared after adjustment for BMI. BMI has been identified as a major risk-factor for VTE in multiple previous studies [12, 23–26], and confounded associations assessed in this study. BMI possibly explains previous positive associations between cardiovascular risk-factors and VTE in studies that were not adjusted for BMI. Studies of genetic variations using Mendelian randomisation provide evidence of the causal link between increased weight and VTE [27, 28]. These results suggest that people with metabolic conditions such as hypertension, diabetes and dyslipidaemia may not be at increased risk of VTE, given that they have a BMI within the healthy range.

VTE commonly presents with comorbid conditions such as endothelial dysfunction [29], and signs of atherosclerosis [30]. Prospective studies into risk factors for VTE provided conflicting results. A 2017 meta-analysis of prospective studies identified an inverse association between systolic blood pressure and VTE risk [11]. This inverse association was strongest in the highest range of systolic pressure (140–200 mmHg), but considering participants who may be approaching hypertensive crises (> 180 mmHg) may not represent the general population, and could be affected by bias due to unmeasured confounding from hypertension medications, lifestyle changes to reverse hypertension, diagnosis biases, or specific surveillance for cardiovascular conditions. Another meta-analysis in 2016 of prospective and case-control studies has indicated that hypertension is associated with an increased risk for VTE compared to non-hypertensive subjects [31]. In the Nurses’ Health Study [32], hypertension was identified as a risk factor for VTE, with a relative risk of 1.9; however, hypertension was not identified as risk factor in the Framingham Heart Study [33]. Our study of French women observed no associations between hypertension and all-VTE, but a weak association between hypertension and PE was identified. It is possible that these results could be explained by uncontrolled confounding from conditions such as pulmonary disease (or other respiratory conditions), which could be associated with both hypertension [34], and the risk of PE [35]. Similarly, isolated PE has been associated with a higher risk of arterial thrombotic events [36], which could possibly be explained by underlying hypertension among people with PE as reported in our results.

Previous studies have mostly shown that neither diabetes nor dyslipidaemia are associated with the risk of VTE [24, 26, 37]. The LITE study identified a slightly increased risk of VTE amongst participants with diabetes (HR = 1.5 (95% CI: 1.0–2.1)) [25]. One meta-analysis [38] of 19 prospective cohort and case control studies found a weak positive, but non-significant association between diabetes and VTE risk (HR = 1.10 (95% CI: 0.94–1.29)). The authors conclude that diabetes is unlikely to play a major role in VTE development, which our study supports. We were however limited by the number of cases which occurred amongst diabetic women, and the relatively low number of diabetic women in the cohort.

We observed a weak association between dyslipidaemia and the risk of primary VTE. Despite controlling for statins, it is possible that this relationship could be confounded by treatment for dyslipidaemia other than statins, or for behavioural changes provoked by a diagnosis of dyslipidaemia. These associations were not consistent when considering self-reported dyslipidaemia cross-referenced with statin reimbursement. Interestingly Wattanakit et al. [12] also observed a weak negative association between high concentrations of LDL-C, and all VTE (HR _LDL > 160_ = 0.73 (0.54: 0.98)), and the UK biobank study observed inverse associations between apolipoprotein B, lipoprotein(a), and the risk of VTE [9]. Further research should be conducted to further understand these observations, which could possibly be resulting from bias after adjusting on an intermediate variable during follow-up, or due to time-varying confounding which may not be appropriately controlled for under the modelling strategy used [39]. It is unclear whether any independent protective effect from statins exists, despite Rovustatin having been shown to reduce the likelihood of VTE in a large RCT [40]. Statins are theorised to reduce the risk of VTE through mechanisms independent to the lowering of LDL-C [41], such as increased anticoagulant activity. Elevated or oxidised low density lipoprotein (LDL) can promote thrombin formation [42], thus an imbalance in lipids may result in a hyper-coagulative state.

In this study, we observed no association between smoking, and the risk of VTE. This is in opposition to previous studies [43, 44] including the large UK biobank study that observed convincing associations between smoking and the risk of VTE [9]. A previous meta-analysis [7] of 32 observational studies has shown that smoking is associated with an increased risk of VTE, with a risk ratio of 1.19 as well as linear responses from pack-years and cigarettes per day. We were unable to reproduce this observation perhaps due to the low number of smokers in this cohort, and that many participants quit smoking during follow-up. It is also possible that the effect of smoking could be mediated, or masked, by a lower BMI among smokers.

Total physical activity as estimated by METS was not associated with the overall risk of VTE, although the highest levels of physical activity were associated with a reduced risk of primary VTE. Previous prospective studies have shown similar results regarding all VTE, and data from the Nurses’ Health Study [45] have shown that time spent inactive (but not total physical activity) is associated with an increased risk of pulmonary embolism. Physical activity could reduce the risk of VTE by improving circulation and the quality of blood-vessels, and lowering the likelihood of blood stagnation in the lower extremities which can lead to deep vein thrombosis. It is possible that time spent stationary could be a more important predictor of VTE than total physical activity.

Regarding ABO group, associations between hypertension and primary VTE were higher within the O group. The AB blood group is associated with an increased risk for VTE, through increased von Willebrand factor (VWF) [46]. Hypertension is also associated with increased VWF concentrations [47], and the O-group is associated with higher rates of hypertension [19]. It is possible that patients with O blood group and hypertension have similar VWF levels as patients with AB blood group and no hypertension, and that further increases in VWF in AB/hypertensive cases are limited. It is less clear why dyslipidaemia appeared associated with a reduced risk only amongst O-group participants, and these observations may have occurred by chance as the *p*-values were non-significant.

### Strengths and limitations

This study has a number of strengths, notably a long follow-up, large number of participants and cases, control for a large set of potential confounders, detailed reporting on the type of VTE, and a validation procedure used for all cases. Exposures of metabolic conditions were verified against specific treatment reimbursements. Regarding hypertension and diabetes, self-reported hypertension showed strong correlation with reimbursements for blood-pressure lowering medications, and diabetes cases were validated via a specific questionnaire. Measured blood pressure values, and cholesterol values were not available for the majority of study participants, thus it was not possible to determine if there is some non-linear relationship between blood-pressure levels/serum cholesterol and the risk of VTE. The dichotomisation of these variables should be considered a major limitation of the current approach.

As later VTE cases were based on self-reports verified by drug reimbursement, it is possible that a number of cases were misdiagnosed, or that cases were missed. Regarding DVT, it is possible that misclassification between superficial and deep thrombosis could occur. Baseline dyslipidaemia cases were based on self-reports, but later cases were verified by treatment reimbursements in secondary analysis. Smoking data was based on self-reports, and included a low number of current smokers, resulting in a lower statistical power to investigate associations among smokers. Similarly, physical activity data was based on self-report and may be subject to error. However, these data were assessed prior to the VTE event, and any error should be independent of the event.

A major strength is the consideration of risk-factors as time dependent variables, as a large number of participants developed hypertension and dyslipidaemia during follow up. However, this method can also introduce bias, through the inclusion of time-varying covariates or potential mediators in the model [39, 48]. Future studies should address this limitation using g-methods [49], which can take into account time-varying confounding without potentially introducing bias from time-varying covariates. Due to this possibility, models considering only baseline data were considered in sensitivity analysis, and showed no major differences. Limitations include the lack of data on medications at study baseline, and the fact that later cases of VTE were validated only via drug reimbursements, and not on information from imaging procedures. Reassuringly, we did observe similar results when only considering cases strictly validated via specific mailings to medical practitioners. The E3N cohort is rather homogenous, and the majority of participants are at a low-risk of non-communicable diseases such as VTE, and cardiovascular disease, thus it is unclear how these observations may translate to higher-risk populations.

As treatments for hypertension, diabetes, and dyslipidaemia are commonly long-term [50], we are confident that these exposures are true representations of these metabolic conditions. Although these conditions are potentially reversible with weight loss and changes in the diet, this would be hard to verify. It is possible that some residual confounding from other lifestyle factors could drive some of the associations observed, especially for the negative associations between dyslipidaemia and primary VTE. The modelling approach chosen, i.e. not mutually adjusting for all considered risk-factors reduces the likelihood of including mediators as in the model, which could introduce collider bias (for example baseline physical activity may influence the future risk of hypertension). Unmeasured confounding is another major concern. For example, certain auto-immune conditions such as rheumatoid arteritis are associated with hypertension [51], and with the risk of VTE [52]. Assuming that these types of conditions can cause hypertension, then associations between hypertension and VTE may be positively biased, which could potentially explain the observations between hypertension and PA. However as these conditions are relatively rare in the general population, we believe that this is a low risk of bias. Similarly, medications that can provoke VTE were unadjusted for, but unless they are causally related to the considered exposures and the thrombotic events, then they should not majorly bias the results. The lack of control for factors such as these is a common limitation in many previous cohort studies of VTE [12, 25].

## Conclusion

Altogether these results suggest that the common risk-factors for CVD are not risk factors for VTE. Regular physical activity may reduce the risk of primary VTE. We confirm previous observations linking dyslipidaemia to a reduced risk of VTE, which should be investigated in future studies. People with metabolic conditions, but with a healthy BMI may not be at higher risk of VTE.

## Supplementary Information

**Additional file 1.**

## Data Availability

The datasets generated and/or analysed during the current study are not publicly available due to data sharing protocols but are available from the corresponding author on reasonable request.

## Abbreviations

CVD: Cardiovascular disease
VTE: venous thromboembolism
PE: pulmonary embolism
DVT: deep vein thrombosis
E3N: Etude Epidémiologique auprès de femmes de la Mutuelle Générale de l’Education Nationale
MGEN: Mutuelle Générale de l’Education Nationale
MHT: menopausal hormone therapy
BMI: body mass index
