# Supplementary material for: Association between cardiovascular risk-factors and venous thromboembolism in a large longitudinal study of French women
Source: Thromb J. 2021 Aug 21;19:58. doi: 10.1186/s12959-021-00310-w (PMC8380360; doi:10.1186/s12959-021-00310-w)

Supplement

Supplementary table 1: Sensitivity analysis adjusting on incident fractures, heart attack, cancer and stroke. Hazard ratio and 95 % confidence intervals for cardiovascular risk-factors and the risk of VTE, and VTE subtypes.

|  | All VTE | |  | Pulmonary embolism | |  | DVT | |  | Primary | |  | Secondary | |
| --- | --- | --- | --- | --- | --- | --- | --- | --- | --- | --- | --- | --- | --- | --- |
|  | Cases | adjusted HR* |  | Cases | adjusted HR* |  | Cases | adjusted HR* |  | Cases | adjusted HR* |  | Cases | adjusted HR* |
| physical act T1 | 545 | ref |  | 166 | ref |  | 376 | ref |  | 163 | Ref |  | 382 | Ref |
| PA T2 | 545 | 1.02 (0.90: 1.15) |  | 160 | 1.04 (0.84: 1.30) |  | 387 | 1.03 (0.89: 1.19) |  | 156 | 0.98 (0.78: 1.22) |  | 389 | 1.06 (0.92: 1.22) |
| PA T3 | 559 | 1.02 (0.91: 1.16) |  | 179 | 1.01 (0.81: 1.25) |  | 381 | 1.01 (0.88: 1.17) |  | 113 | 0.71 (0.56: 0.91) |  | 446 | 1.14 (1.00: 1.31) |
|  | | | | | | | | | | | | | | |
| Never smoker | 946 | ref |  | 295 | ref |  | 652 | ref |  | 253 | ref |  | 693 | ref |
| X smoker | 557 | 0.95 (0.86: 1.06) |  | 166 | 0.94 (0.78: 1.14) |  | 392 | 0.94 (0.83: 1.07) |  | 131 | 0.84 (0.68: 1.04) |  | 426 | 0.81 (0.66: 1.01) |
| smoker | 146 | 0.87 (0.73: 1.03) |  | 44 | 0.96 (0.70: 1.33) |  | 100 | 0.84 (0.68: 1.03) |  | 48 | 0.99 (0.73: 1.36) |  | 98 | 0.96 (0.88: 1.12) |
|  | | | | | | | | | | | | | | |
| hypertension | 827 | 1.05 (0.95: 1.16) |  | 296 | 1.20 (1.00: 1.45) |  | 532 | 0.99 (0.88: 1.12) |  | 204 | 1.02 (0.84: 1.26) |  | 623 | 1.07 (0.95: 1.21) |
| dyslipidaemia | 354 | 0.90 (0.79: 1.01) |  | 142 | 1.03 (0.84: 1.26) |  | 212 | 0.83 (0.71: 0.97) |  | 69 | 0.68 (0.52: 0.89) |  | 285 | 0.97 (0.85: 1.12) |
| diabetes | 36 | 0.73 (0.52: 1.02) |  | 17 | 0.88 (0.54: 1.44) |  | 19 | 0.62 (0.40: 0.99) |  | 11 | 0.95 (0.52: 1.75) |  | 25 | 0.66 (0.44: 0.99) |

*adjusted on education level, statin use, menopause, MHT use, parity, type of menopause, family history of CVD, BMI, incident fractures, stroke, cancer and myocardial infarction

Supplementary figures


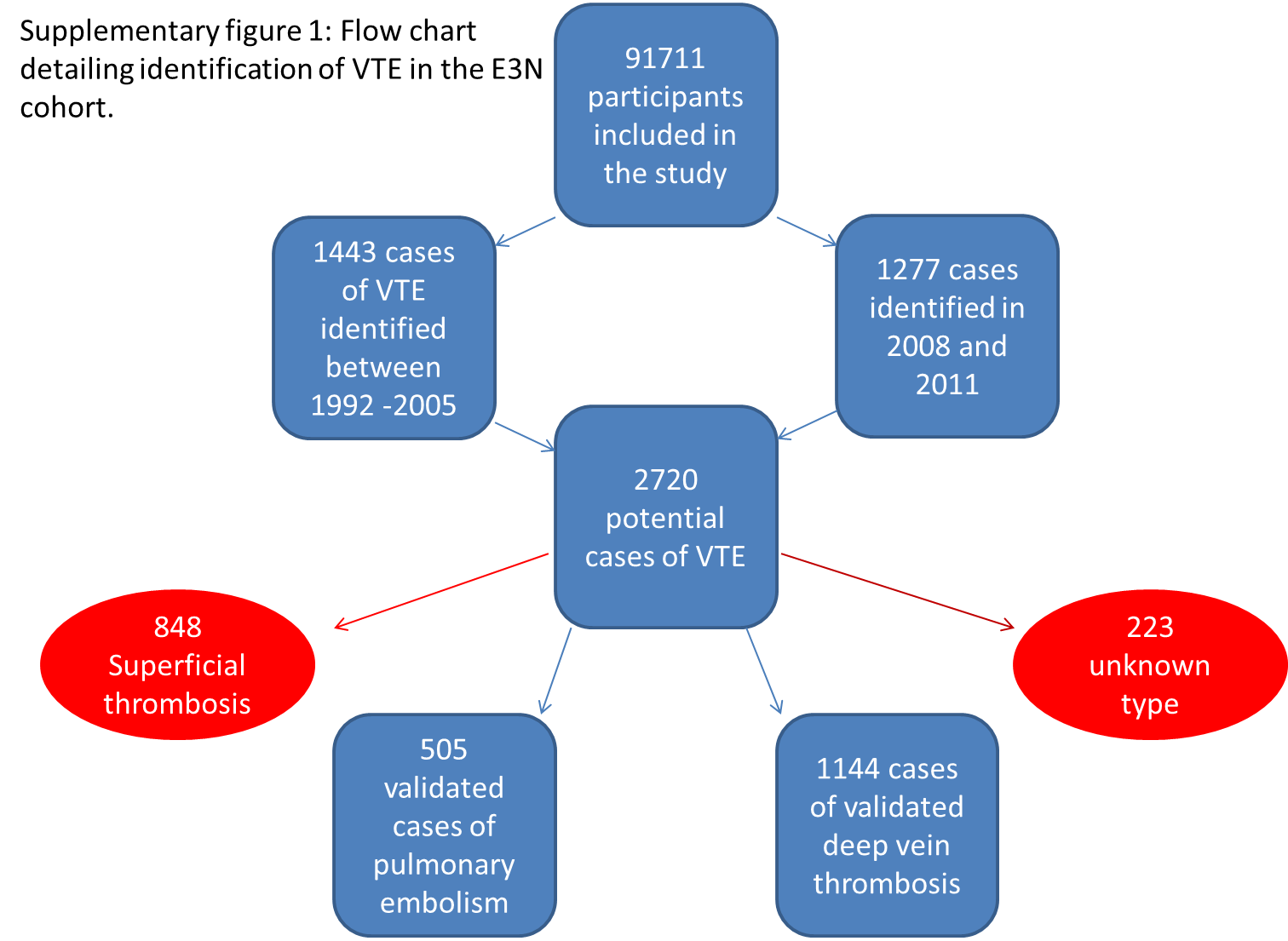


Supplementary Figures

S Figure 2: Causal diagrams showing assumed relations between cardiovascular risk-factors and VTE

1. Cardiometabolic factors including diabetes, hypertension and dyslipidaemia


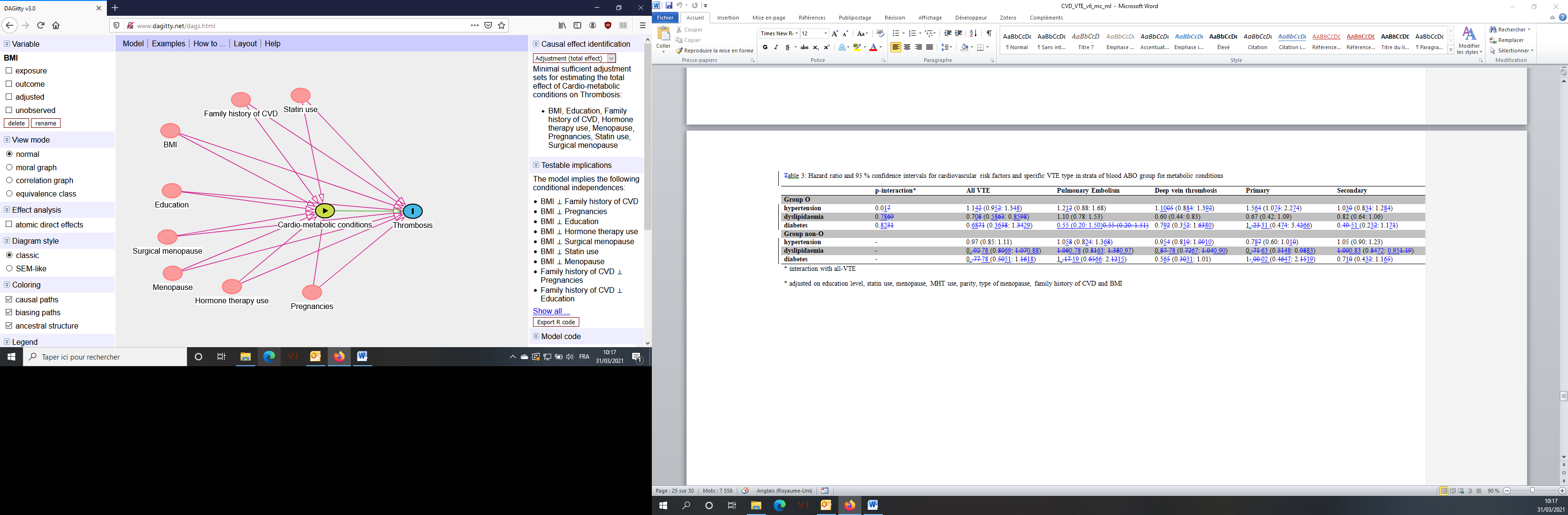


1. Physical activity


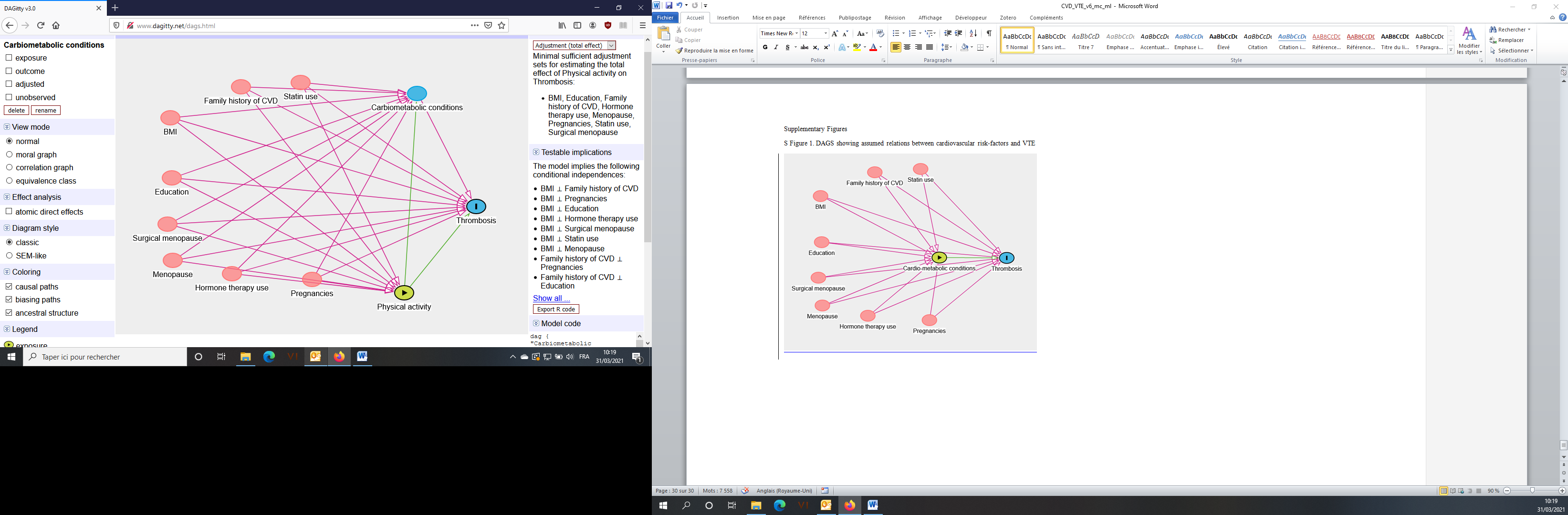


1. smoking


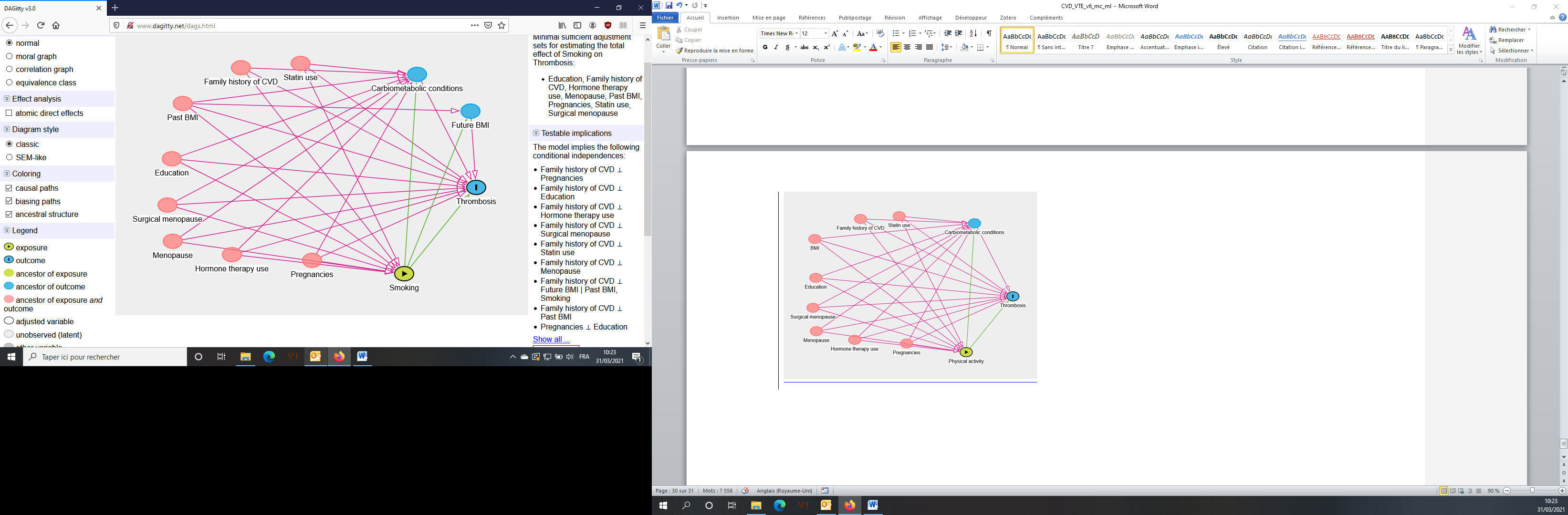

Supplement: Supplementary file 1 — Additional file 1. [file 12959_2021_310_MOESM1_ESM.docx]
